# Formic acid sandwich method is well-suited for filamentous fungi identification and improves turn around time using Zybio EXS2600 mass spectrometry

**DOI:** 10.1186/s12866-024-03394-2

**Published:** 2024-07-03

**Authors:** Chongyang Wu, Keping Ao, Yue Zheng, Ying Jin, Ya Liu, Zhixing Chen, Dongdong Li

**Affiliations:** 1 Department of Laboratory Medicine, West China Hospital of Sichuan University, Sichuan Province, Chengdu, 610041 P.R. China; 2 Department of Laboratory Medicine, Yaan People’s Hospital, Yaan, 625000 China; 3 Department of Laboratory Medicine, LuZhou Longmatan TCM Hospital, LuZhou, 646000 China

**Keywords:** MALDI-TOF MS, EXS2600, Filamentous fungi, Formic acid sandwich, Mold extraction kit

## Abstract

**Objectives:**

Matrix-assisted laser desorption/ionization time-of-flight mass spectrometry (MALDI-TOF MS) is extensively employed for the identification of filamentous fungi on MALDI Biotyper (Bruker Daltonics) and Vitek MS (biomerieux), but the performance of fungi identification on new EXS2600 (Zybio) is still unknow. Our study aims to evaluate the new EXS2600 system's (Zybio) ability to rapidly identify filamentous fungi and determine its effect on turnaround time (TAT) in our laboratory.

**Methods:**

We tested 117 filamentous fungi using two pretreatment methods: the formic acid sandwich (FA-sandwich) and a commercial mold extraction kit (MEK, Zybio). All isolates were confirmed via sequence analysis. Laboratory data were extracted from our laboratory information system over two 9-month periods: pre-EXS (April to December 2022) and post-EXS (April to December 2023), respectively.

**Results:**

The total correct identification (at the species, genus, or complex/group level) rate of fungi was high, FA-sandwich (95.73%, 112/117), followed by MEK (94.02%, 110/117). Excluding 6 isolates not in the database, species-level identification accuracy was 92.79% (103/111) for FA-sandwich and 91.89% (102/111) for MEK; genus-level accuracy was 97.29% (108/111) and 96.39% (107/111), respectively. Both methods attained a 100% correct identification rate for *Aspergillus*, *Lichtheimia*, *Rhizopus Mucor* and *Talaromyces* species*,* and were able to differentiate between *Fusarium verticillioides* and *Fusarium proliferatum* within the *Fusarium fujikuroi* species complex. Notably, high confidence was observed in the species-level identification of uncommon fungi such as *Trichothecium roseum* and *Geotrichum candidum*. The TAT for all positive cultures decreased from pre EXS2600 to post (108.379 VS 102.438, *P* < 0.05), and the TAT for tissue decreased most (451.538 VS 222.304, *P* < 0.001).

**Conclusions:**

The FA-sandwich method is more efficient and accurate for identifying filamentous fungi with EXS2600 than the MEK. Our study firstly evaluated the performance of fungi identification on EXS2600 and showed it is suitable for clinical microbiology laboratories use.

**Supplementary Information:**

The online version contains supplementary material available at 10.1186/s12866-024-03394-2.

## Highlights

(1) The EXS2600 platform demonstrated superior capability in distinguishing between *Fusarium* species.

(2) The data demonstrated that EXS2600 platform provided comparable results and was suitable for filamentous identification.

(3) The EXS2600 platform significantly reduced the TAT for the processing of positive cultures in tissue specimens.

**Supplementary Information:**

The online version contains supplementary material available at 10.1186/s12866-024-03394-2.

## Introduction

In recent years, the employment of MALDI-TOF MS for the identification of bacteria and yeasts has increased significantly due to its remarkable accuracy. However, the identification of molds and fungi remains one of the most challenging aspects of microbiology [1]. The accuracy of identifying filamentous fungi is varied by MALDI-TOF MS, with a range from 72 to 90% [2, 3]. Currently studies have employed Bruker Microflex or the biomerieux Vitek MS to classify filamentous fungi [2, 4], bu the existing libraries' limitations or less ideal spectrum database displayed that over 20% of fungi could not be pinpointed to the species-level when using Bruker Microflex databases [5], and the biomerieux Vitek MS failed to recognize 10.5% of specimens [6]. The results of incorrect identification were interpreted differently depending on the presence of species in the database, which is a major factor influencing the MALDI-TOF MS performance [7]. The expanded database of Zybio MALDI-TOF MS EXS2600 (Chongqing, People’s Republic of China) includes different fungi spectra, which have been generated through various pretreatment methods and growth periods. On the other hand, some fungal species exhibited unsatisfactory peak fingerprints in mass spectrum based the MS identification. These suboptimal peaks lead to false positive results when using reduced quality or quantity standard fingerprints. In order to improve the accuracy identification of fungi, the Zybio’ database is divided into the Common Clinical Database and Special Fungi Database. The clinical database retains well-characterized fungal strains that are commonly encountered in clinical settings with rich peak fingerprints, and are less prone to confusion. The special database stores a broader range of fungal species and maximizes the detection rate for less common fungi.

In addition to the limitations of database affecting identification accuracy, protein extraction presented another challenge for the identification of filamentous fungi. Difficulty in breaking walls to obtain low quality spectra. And the complexity protein fingerprints of filamentous fungi at different growth cycle periods could be another problem. Usually, filamentous fungi form a large number of hyphae on solid medium, and the conidia are difficult to lysis [8]. The most common pretreatment method for filamentous fungi involves the extraction of proteins with formic acid (EtOH-FA full extraction), a process that requires two rounds of centrifugation [9]. The 88.70% of clinical isolates were identified to the species level, using this method with the in-house spectra library created by Becker et al. [9]. Such complicated pretreatment method hampered the application of MALDI-TOF MS for identifying fungi and time-consuming [10]. Dan Peng et al. described an alternative method, the FA-sandwich, which allowed a 93.9% species-level identification with Autof MS, and 97.3% species-level identification rates were found for Vitek MS when IVD and in-house database combination [11]. Moreover, there were some commercial protein extraction kits for the pretreatment of fungal identification, the commercial VITEK MS Mould Kit (bioMerieux, France) was used and 89.0% of the isolates were correctly identified by VITEK MS system [12]. In the present study, the performance of two fast pretreatment methods (MEK and FA-sandwich) for filamentous fungi identification were evaluated using Zybio MS EXS2600, and the TAT of positive-culture clinical specimens between pre and post EXS2600 was compared.

To address the issue of poor repeatability in filamentous fungi identification, a comprehensive library comprising mass spectra from filamentous fungi at various stages of culture has been constructed, which obtained complete mass fingerprinting covering each strain. For instance, the mass spectra of *Aspergillus candidus* and *Lichtheimia corymbifera* after three days of culture were similar to those obtained seen after seven days. To reduce false matches, the three-day cultures spectra were included in the library. Conversely, for *Penicillium oxalicum* and *Trichoderma koningii,* the seven-day cultures provided different spectra from the three-day ones, so we added the seven-day spectra to the library as well.

Furthermore, the system constructed a library of filamentous fungi spectra using various pretreatment methods, including EtOH-FA full extraction, MEK and FA-sandwich [11]. In summary, the thoughtful design of the intelligent search algorithm has contributed to the Zybio EXS2600 system’s effectiveness in identifying filamentous fungi.

Our findings displayed that both methods have high identification rates for clinical filamentous fungi and distinguish complex species like *Fusarium*. The FA-sandwich method is well-suited for filamentous fungi identification with EXSW2600, leading to significant improvements in TAT in clinical laboratories.

## Materials and methods

### Sample collection and species identification

In this study, clinical fungal strains were isolated from patients with fungal infections at the Department of Laboratory Medicine, West China Hospital, Sichuan University (Jan 2019 to Nov 2023) and all of the isolates were stored at -80℃. To assess the performance of EXS2600 system in identifying filamentous fungi, those strains were recovered on the Sabouraud’s dextrose agar plates (AutoBio, China) and incubated at 28℃ for 2–5 days. The fungal hyphae were identified by lactophenol cotton blue staining when it was suitable for morphological identification. A total of 117 filamentous fungi were collected after excluding non-activated or contaminated strains (Tables 1 and 2). Each isolate was tested for one time with four biological replicates by EXS2600. All strains were subjected to molecular sequencing as a reference. Sanger sequencing for the internal transcribed spacer (ITS) regions with ITS1/ITS4 or 18S rRNA genes was carried out as primary species level identification [13]. Sequencing of the translation elongation factor 1-α gene (TEF-1α) and RNA polymerase II gene (rPB2) was conducated for *Fusarium* species [14]. Sequences were analyzed using the MycoBank (https://www.mycobank.org) and the National Center for Biotechnology Information Nucleotide BLAST (NCBI, https://blast.ncbi.nlm.nih.gov/Blast.cgi) databases, and the results were accepted if identity > 98% with > 95% query coverage.

**Table 1:** Identification of clinical filamentous fungi by EXS2600 using the FA-sandwich and MEK methods

**Species**	**No. of isolates**	**Data for FA-sandwich method**				**Data for MEK method**			
		**Species**	**Genus **	**No ID**	**Mis ID**	**Species**	**Genus**	**No ID**	**Mis ID**
***Aspergillus***	**9**								
* A. calidoustus*	1	1				1			
* A. clavatus*	1	1				1			
* A. neoniveus*	1	1				1			
* A. niger*	2	2				2			
* A. terreus*	3	3				3			
* A. versicolor*	1	1				1			
***Cunninghamella***	**5**								
* C. bertholletiae*	3	3				3			
* C. echinulata*	2	2				2			
***Exophiala***	**6**								
* E. dermatitidis*	5	5				5			
* E. jeanselmei*	1			1				1	
***Fusarium***	**17**								
* F. delphinoides*	2	2				2			
* F. fujikuroi *	1				1				1
* F. incarnatum*	1	1				1			
* F. oxysporum*	1	1				1			
* F. keratoplasticume*	1	1				1			
* F. proliferatum*	2	2				2			
* F. solani*	7	7				7			
* F. verticillioides*	2	2				2			
***Lichtheimia***	**6**								
* L. corymbifera*	4	4				4			
* L. ramosa*	2	2				2			
***Mucor***	**11**								
* M. circinelloides*	7	7				7			
* M. hiemalis*	1	1				1			
* M. irregularis*	1	1				1			
* M. racemosus*	1	1				1			
* M. ramosissimus*	1	1				1			
***Rhizopus***	**12**								
* R. arrhizus*	4	4				4			
* R. microsporus*	6	6				6			
* R. pusillus*	1	1				1			
* R. stolonifer*	1	1				1			
***Scedosporium***	**4**								
* S. apiospermum*	2	2				2			
* S. boydii*	1	1				1			
* S. dehoogii*	1				1				1
***Sporothrix schenckii***	**6**	6				6			
***Talaromyces***	**13**								
* T. amestolkiae*	1	1				1			
* T. marneffei*	12	12				12			
***Trichoderma***	**3**								
*** T. longibrachiatum***	2	2				2			
*** T. harzianum***	1		1				1		
***Trichophyton***	**11**								
* T. interdigitale*	5	1	4			1	4		
* T. mentagrophytes*	4	4				4			
* T. rubrum*	1	1				1			
* T. tonsurans*	1	1				1			
**Rare species**	**9**								
* Alternaria alternata*	1	1				1			
* Cladosporium sphaerospermum*	1	1				1			
* Geotrichum candidum*	1	1				1			
* Microsporum gypseum*	1	1				1			
* Penicillium singorense*	1	1				1			
* Purpureocillium lilacinum*	1	1				1			
* Scopulariopsis candida*	1	1				1			
* Syncephalastrum racemosum*	1	1						1	
* Trichothecium roseum*	1	1				1			
**Total**	111	103	5	1	2	102	5	2	2

*No ID* no identification result, *Mis ID* mis identification result

**Table 2:** Analysis of the genus-level, no identification or mis identification filamentous fungi strains in this study

Strain	Molecular identification	Reference spectrum in database	FA-sandwich	Score	MEK	Score
MS_09	*Irpex lacteus*	NO	*Irpex* species	1.78	NO-identification	/
MS_21	*Pestalotiopsis chamaeropis*	NO	NO-identification	/	NO-identification	/
MS_43	*Exserohilum rostratum*	NO	*Curvularia clavata*	1.56	*Curvularia clavata*	1.58
MS_28	*Fusarium falciforme*	NO	*Fusarium solani complex*	2.39	*Fusarium solani complex*	1.81
MS_35	*Fusarium falciforme*	NO	*Fusarium solani complex*	2.25	*Fusarium solani complex*	1.73
MS_40	*Fusarium falciforme*	NO	*Fusarium solani complex*	1.67	*Fusarium solani complex*	1.87
MS_12	*Scedosporium dehoogii*	YES	*Scedosporium apiospermum*	1.89	*Scedosporium apiospermum*	2.12
MS_91	*Trichoderma harzianum*	YES	*Trichoderma* species	1.88	*Trichoderma* species	1.7
MS_32	*Fusarium fujikuroi*	YES	*Fusarium proliferatum*	1.88	*Fusarium proliferatum*	1.79
MS_01	*Syncephalastrum racemosum*	YES	*Syncephalastrum racemosum*	1.86	NO-identification	/
MS_68	*Exophiala jeanselmei*	YES	NO-identification	/	NO-identification	/
MS_72	*Trichophyton interdigitale*	YES	*Trichophyton mentagrophytes complex*	1.89	*Trichophyton mentagrophytes complex*	2.01
MS_73	*Trichophyton interdigitale*	YES	*Trichophyton mentagrophytes complex*	1.99	*Trichophyton mentagrophytes complex*	1.89
MS_74	*Trichophyton interdigitale*	YES	*Trichophyton mentagrophytes complex*	2.01	*Trichophyton mentagrophytes complex*	1.87
MS_75	*Trichophyton interdigitale*	YES	*Trichophyton mentagrophytes complex*	1.79	*Trichophyton mentagrophytes complex*	1.95

Scores represent the confidence level of identification, with higher scores indicating better matches to reference spectra. This system indicated the reliability of identification results by a color-coded categorization, green results (score: ≥2) represent measurements valid at the species level and yellow results (score: 1.70-1.99) represent measurement valid at the genus level

### Protein extraction and MALDI-TOF MS system scoring scheme

**Fig. 1:**
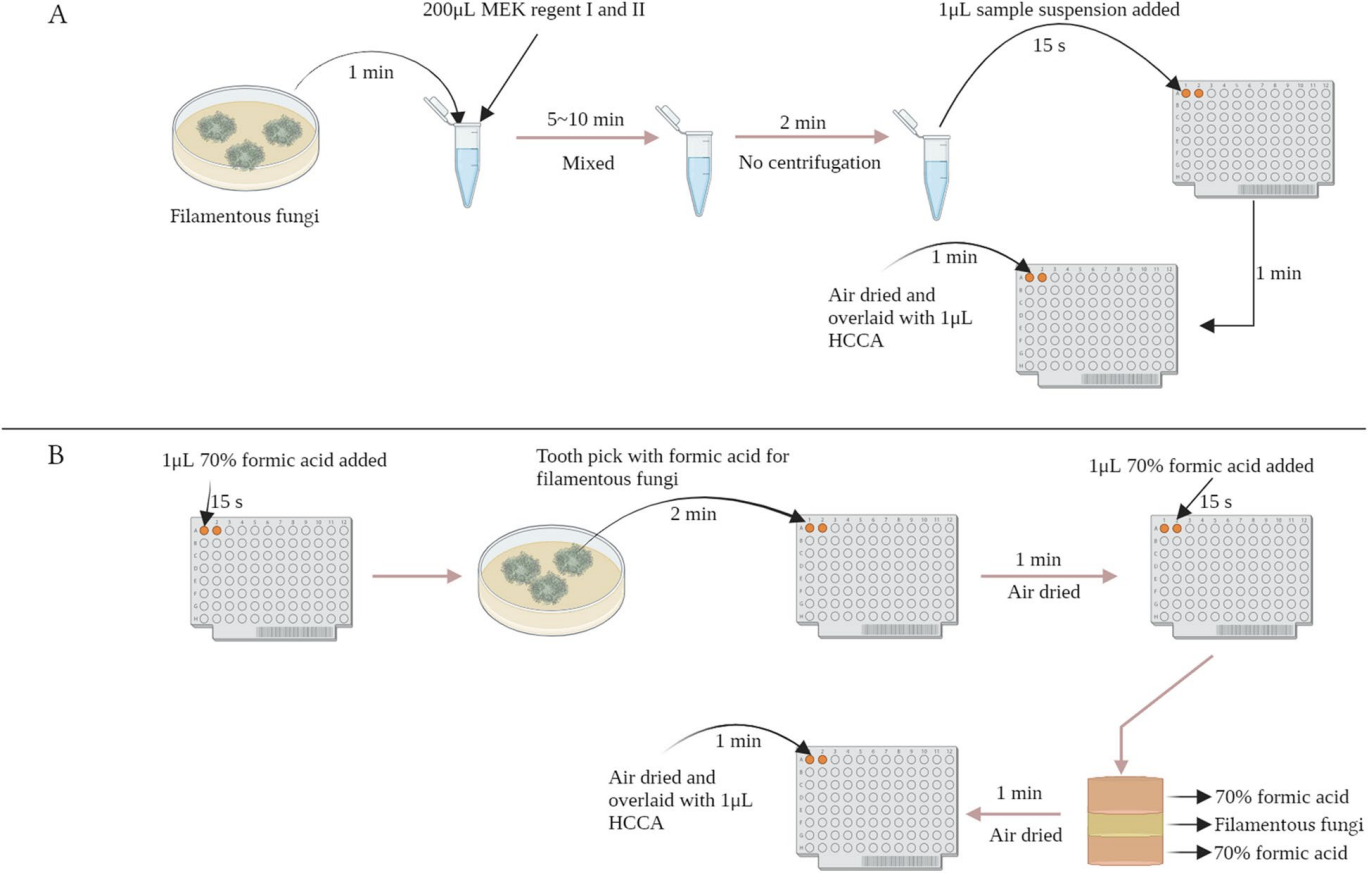
The illustration of the MEK and FA-sandwich pretreatment methods. **A** The MEK method: i) approximately three colonies in 200μL mold extraction regent I and 200μL regent II in a 1.5 mL Eppendorf tube (1 min); ii) the mixture was vortexed and mixed (5 ~ 10 min), and then no centrifugation (2 min); iii) the 1μL of samples suspension was spotted on the target plate (15 s), air dried (1 min) and overlaid with 1μL of α-Cyano-4-hydroxycinnamic acid (HCCA) matrix solution (1 min); iv) the plate was dried at room temperature again and then placed into the instruction for testing. **B** The FA-sandwich method: i) 1μL 70% formic acid was applied onto an MBT biotarget 96 (15 s); ii) toothpick is dipped into a droplet of formic acid for obtaining fungi’s front mycelium, instantly enabling reaction of the acid with the fungi (2 min); iii) the fungi subsequently smeared in the 70% formic acid droplet on the target (15 s) and formed a (formic acid: mycelia: formic acid) sandwich; iv) air dried (1 min) and overlaid with 1μL of HCCA matrix solution and air dried again (1 min), then for the identification

Two protein extraction assays were used in parallel (Fig. 1). One is MEK recommended by the manufacture; the other is FA-sandwich*.* The isolates were used for a comparison of Zybio’s EXS2600 with the “v3.0.2.2” database.

### Specimens TAT analysis of filamentous *fungi* isolates

To evaluate the impact of EXS on overall TAT in routine practice, we exported the detailed information to analysei the TAT of cultures. Data collection included the detailed information on TAT, species types, identification results of all positive cultured samples, and they were recorded by our Laboratory Information System (Xinhe, Shanghai, China). Laboratory data for all fungal cultures were extracted from our laboratory information system in two 9-month periods: pre-EXS (April to December 2022) and post-EXS (April to December 2023), respectively. Through standard laboratory procedures for cultivation and identification, pre-EXS followed the traditional morphological identification process, while post-EXS using the Zybio identification process. TAT for samples was compared between pre and post periods using the Mann–Whitney U test for analysis. All statistical analyses conducted were two-tailed, with* P* values < 0.05 deemed to indicate significance.

## Results

### Validity of results for two methods of FA-sandwich and MEK

Initially, we successfully recovered a total of 117 filamentous fungi for mass spectrometry analysis (Tables 1 and 2). The total correct identification (at the species, genus, or complex/group level) rates of fungi were high, FA-sandwich (95.73%, 112/117), followed by MEK (94.02%, 110/117). Excluding four species (six isolates) without reference spectra in the database, including *Irpex lacteus* (*n*=1), Pestalotiopsis chamaeropis (n=1), Exserohilum rostratum (*n*=1),*Fusarium falciforme (n=3)*. The correct identification rates of EXS2600 using FA-sandwich were 92.79% (103/111) in species level and 97.29% (108/111) in genus level. The successful identification rates of the MEK were 91.89% (102/111) in species level, and 96.39% (107/111) in genus level. The accuracy of both assays was 100% for *Cunninghamella*, *Lichtheimia* and *Scedosporium* species. There was no significant difference in the accuracy of filamentous fungi identification among different extraction methods on EXS2600 platform.

### Characterization of mis-identified, no-identified, complex species and rare isolates

No identification results were produced by FA-sandwich method for 2 isolates: *P. chamaeropis* and *Exophiala jeanselmei*, by MEK method for 4 isolates: *I. lacteus, P. chamaeropis*, *E. jeanselmei* and *Syncephalastrum racemosum*. Analyzing the results of mis identification of filamentous fungi, *Exserohilum rostratum, Scedosporium dehoogii* and *Fusarium fujikuroi* were mis identified by using both methods. *S. dehoogii* and *F. fujikuroi* included in the database were mis-identified to as “*S. apiospermum* and *F. proliferatum*” using both two methods. *E. rostratum* was not included in the reference data and was mis identified as *Curvularia clavata* by these two methods.

The outcome of identification of *Fusarium* species and rare filamentous fungi showed that highly validation performance was observed when using these two methods in EXS2600. For the *Fusarium* species, the EXS2600 showed good ability to identify *F. solani* (100%, accuracy rate by two methods), and could accurately identify *F. verticillioides* and *F. proliferatum*. In addition, *F. falciforme* was not represented in the database, and as a result, it could only be identified at the *Fusarium solani complex* level. For the relatively rare filamentous fungi isolates, such as *Cladosporium sphaerospermum, Purpureocillium lilacinum,* the species were identified with 100% accuracy by EXS2600 with the FA-sandwich and MEK.

### Comparison of TAT of positive-culture clinical specimens between pre and post EXS2600.

**Fig. 2:**
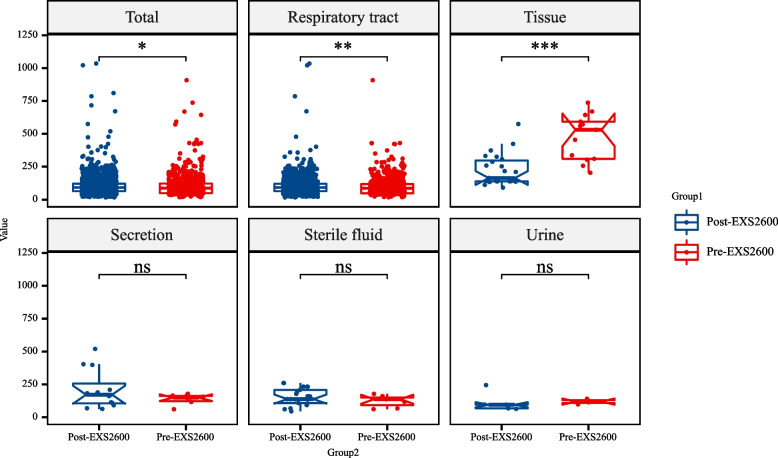
Comparison of TAT of all filamentous fungi positive-culture clinical specimens before and after using EXS2600 system. ***P < 0.001; **P < 0.01; *P < 0.05; ns, no significance

A total of 2023 positive-culture specimens were analyzed, with 604 and 1419 filamentous fungi isolated from the pre- and post- EXS2600, respectively (Fig. 2). Identification of filamentous fungi with EXS2600 revealed significant time saving compared to pre-MALDI-TOF MS. For all types of positive cultures, the TAT was significant decreased between the pre- and post- EXS2600 periods and the TAT of tissue decreased the most (451.538 VS 222.304, *P* < 0.001). Pathological tissue samples cultured for filamentous fungi yielded a variety of species, including *Aspergillus fumigatus* and *Aspergillus flavus*, *Trichophyton rubrum*, *F. solani*, and *Sporothrix schenckii* (Supplementary material Table 1).

## Discussion

The MALDI-TOF MS has changed the way of microbial identification. The speed, accuracy, use-friendly and cost-effectively of the system, leading the global clinical laboratory recognition it as the rapid microbiology method for classifying microorganism. However, in practical applications, the identification results for filamentous fungi are not optimal, primarily due to the complex steps of protein extraction and the limitations of commercial databases [15]. As we all know, the most common method for the pretreatment of filamentous fungi is the Ethanol-formic-acid-acetonitrile extraction (EtOH-FA full extraction), however, the complicated procedures of this method may not satisfy the needs of the rapid identification in clinical laboratories [16]. We first evaluated the EXS system performance of filamentous fungi with the rapid pretreatment: FA-sandwich assay or MEK. It is noteworthy that, utilizing these two methods in conjunction with the EXS2600 commercial database "v3.0.2.2" and without any modifications to the database. In our study, the FA-sandwich (95.73%, 112/117) assay had better performance than the MEK (94.02%, 110/117) at genus level. Furthermore, excluding six strains that were not included in the database, the correct rate at 92.79% (103/111) in species levels and 97.29% (108/111) in genus level when using FA-sandwich assay, and a previous study also reported that the FA-sandwich method can significantly increase the identification ability on Autof ms (93.9%) and Vitek MS (97.3%) when the IVD, the RUO or in house database are used in combination [11]. Similarly, it has been reported that the FA-sandwich method was more effective than EtOH-FA full extraction for identifying clinically common filamentous fungi, such as *Aspergillus* and *Penicillium*, when using the Vitek, Bruker, and Autof MS systems [17, 18]. In the present study, the pretreatment method of the protein extraction kit also was evaluated, and those results displayed that these two rapid methods had general applicability in most fungi species. However, the mis identification of *E. jeanselmei* and *S. dehoogii* suggests that challenges with protein extraction may be due to the inherent properties of fungal cell walls, impeding accurate fungal identification [8, 19]. Future studies should be evaluated and optimized using different protein approaches for different species.

Besides, for the distinguish of *Fusarium* species, which tend to be multi-resistant and are the second most common filamentous fungi causing invasive fungal infections in immunocompromised patients [20]. Zybio’s mass spectrometry strives to differentiate between the most important *Fusarium* species associated with human disease, such as *F. solani, Fusarium keratoplasticum, Fusarium oxysporum, F. proliferatum* and *Fusarium verticillioides,* etc., as well as rare *Fusarium* species including *Fusarium dimerum, Fusarium delphinoides* and *Fusarium incarnatum*. At the same time, these *Fusarium* species were also incorporated into their respective complex groups in the database based on molecular sequencing, for clearer classification and identification. In our study, our data revealed good applicability with the existing database to classify *Fusarium,* except *F. falciforme* were mis-identified as *F. proliferatum.* Previously studies of Vitek MS and Bruker demonstrated a lower rate of correct identification to the species-level by all methods compared to the present study [12, 21, 22]. Specifically, *F. proliferatum* and *F. verticillioides* could not be distinguished and only be identified as a complex group by VITEK MS [23]. As the previous report suggested, the identification of *Fusarium* by other systems required self-established databases and public databases [24]. At the same time, it should be noted that NCBI database will have 10% error comparison results, and a more professional fungal identification database should be used [25]. In this laboratory, the identification effect of *Fusarium* is much higher than other research results when only commercial databases are used. At the same time, our data also showed that the *Trichophyton interdigitale* were mostly identified as the *Trichophyton mentagrophytes* complex (80%). According to the reference spectrum profiles, the *T. interdigitale* was collectively categorized into a single *T. mentagrophytes* complex with minor differences of clinical significance [26]. On the other hand, we also investigated the performance ability to identify the rare fungi isolated from clinical laboratory. The high accuracy rate (100%) using the FA-sandwich method was observed in our tested 9 rare filamentous fungi. The presence of these rare fungi poses a risk to immunocompromised individuals [27]. Therefore, their accurate and rapid identification could improve the diagnostic capabilities for mycoses in our laboratory, and broadly used in clinical laboratories with limited resources [28].

Our workflow displayed that the use of these two methods without inaction step, and the entire process was conducted within a biosafety cabinet to mitigate the biosafety risks associated with the dispersal of fungal spores. Although there is no significant difference of identification accuracy between the MEK and the FA-sandwich method, the FA-sandwich benefits from its simple steps, rapid protein extraction without reagent cost and acetonitrile, and is more suitable for application in most clinical microbiology laboratories (Table 3) [29]. Traditionally, each filamentous fungi identified by the morphology of spore and hyphal required at least 5–10 days of simple cultured [30]. However, as indicated by Fig. 2, the FA-sandwich method requires only a small amount of fungal sample to be placed on the target plate. This enables the acquisition of identification results even when colonies are very small, suggesting that the FA-sandwich assay can identify filamentous fungi at their early stages of growth (2–5 days). In our study, we found that the use of mass spectrometry to identify positive fungal cultures can significantly reduce TAT time, including a variety of sample types, such as tissue specimen. Our findings also revealed that the filamentous fungi cultured from tissue samples included species like *Sporothrix schenckii*, *Fusarium* species, *Trichophyton*, and *Aspergillus* (Table S1). These fungi typically require an extended period for culturing and morphological identification [31]. However, the TAT was significantly reduced to identify very small colonies by using the EXS2600 system. Furthermore, analysis the capability of the EXS system to identify filamentous fungi revealed that correct identification rates for *Aspergillus*, *Mucor*, *S. schenckii* and *Talaromyces* can be achieved at 100%. Consequently, the identification results obtained via mass spectrometry can be regarded as reliable in our clinical practice. Incorporating this EXS system into our routine workflow has revolutionized the efficiency of our mycological identification processes, and enhanced the accuracy and reduced the time required for mold and fungi identification, thereby diminishing our dependence on traditional phenotypic characteristics [9].

**Table 3:** Comparison of the ease of practice between the FA-sandwich and EtOH-FA full, MEK pretreatment

	FA-sandwich	MEK	EtOH-FA full extraction
Fungi amount	1 mm diameter	10-20 mm diameter	30-40 mm diameter
Operation time	5–10 min/sample	10–20 min/sample	30–40 min/sample
Procedures	lysis directly on plate	vortex	vortex and centrifugation
Centrifugation	No	No	Yes
Equipment and regent	formic acid, toothpick	commercially available kit, 1.5 ml microcentrifuge tubes, toothpick	formic acid, ethanol, 1.5 mL microcentrifuge tubes, vortex oscillator, acetonitrile, toothpick
Positive aspects	convenient, time-saving, suitable for clinical microbiology laboratories	convenient, suitable for clinical microbiology laboratories	comprehensive extraction of proteins
Negative aspects	may not be suitable for all fungal species	requires additional cost for purchasing the kit	time-costing, a large amount of fungi is required, process involving ethanol, formic acid, acetonitrile, and centrifugation

To our knowledge, this is the first study to presenting a parallel comparison of two different pretreatment method in the EXS2600 MALDI-TOF MS device and database. This study validated EXS2600 system for the identification of filamentous fungi by using FA-sandwich method. The sample processing method has been simplified, requiring less reagents, and significantly decreased the TAT of fungi culture-positive specimens in clinical laboratory medicine, demonstrating its usefulness to rapidly identify fungal isolates from clinical samples and applying the optimal fungal treatment accordingly.

This study has some limitations, with regard to the overall low sample size, it is evident that a study based on 117 isolates cannot provide a definitive comparison of the MALDI-TOF MS systems. However, the failure to identify cetain fungi, such as *E. jeanselmei, S. dehoogii* and *F. fujikuroi*, these data do not feature an insight into the lower capability of EXS to support routine diagnostics in a clinical microbiology laboratory. A more comprehensive list of filamentous fungi is needed and filamentous fungi species commonly encountered in other regions should be tested, in order to compare the ability to identify fungi using these methods in either the biomerieux or the Bruker device.

### Supplementary Information

Additional file 1: Supplementary Table S1. Laboratory data for all fungal cultures were collected from laboratory information system in two 9-month periods: pre-EXS (April to December 2022) and post-EXS (April to December 2023).

Additional file 2: Supplementary Figure S1. Mass spectra of five representative filamentous fungi were collected at three and seven days of culture, and peaks with significant differences were marked in red boxes.

## Data Availability

We are submitting the sequences data of all filamentous fungi to NCBI. The direct web link is as follows: https://dataview.ncbi.nlm.nih.gov/object/PRJNA1085986. BioProject ID: PRJNA1085986. After review by the database staff, our BioSample information will be accessible with the link.
