# Supplementary figures and images for: Formic acid sandwich method is well-suited for filamentous fungi identification and improves turn around time using Zybio EXS2600 mass spectrometry
Source: BMC Microbiol. 2024 Jul 3;24:238. doi: 10.1186/s12866-024-03394-2 (PMC11220970; doi:10.1186/s12866-024-03394-2)

A

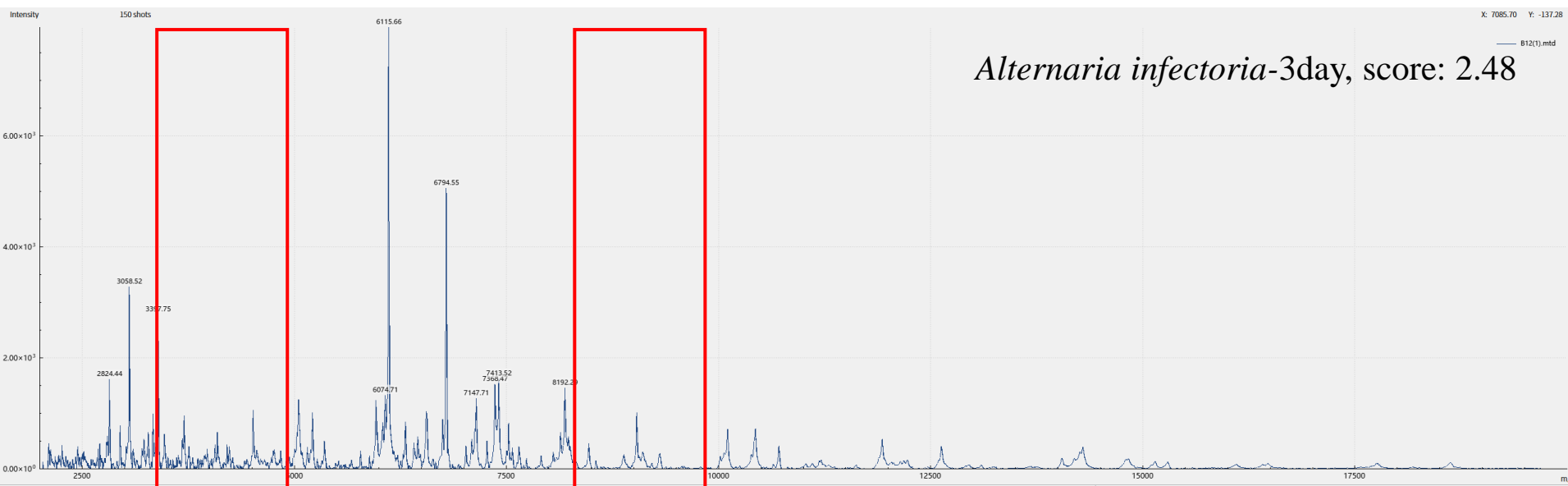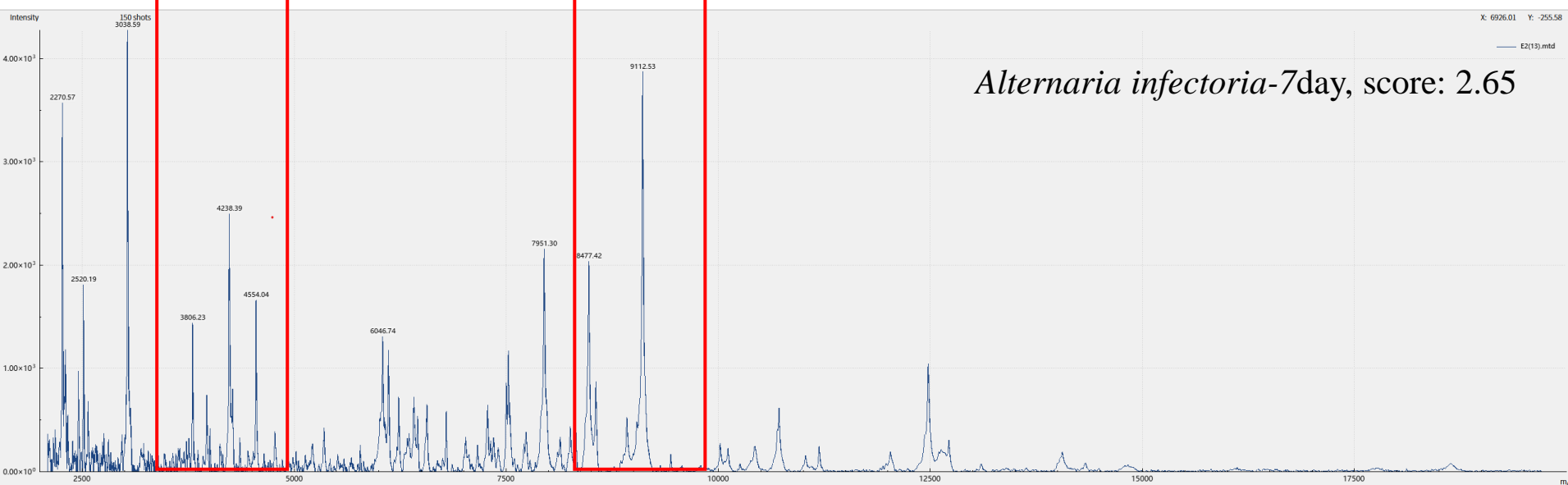

B

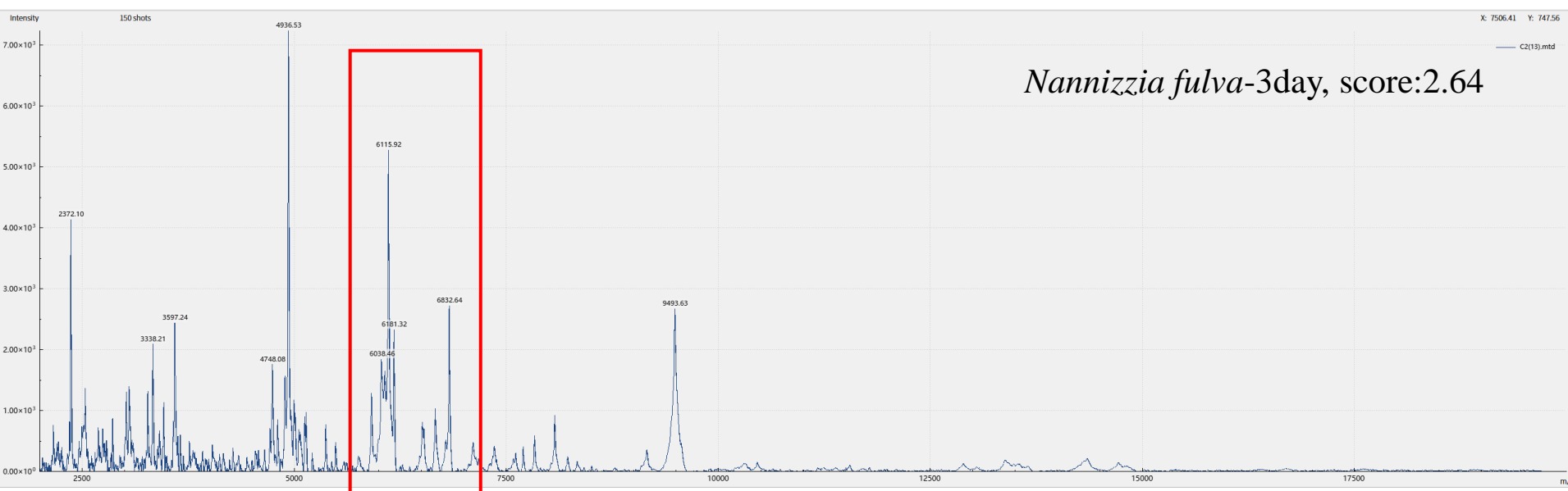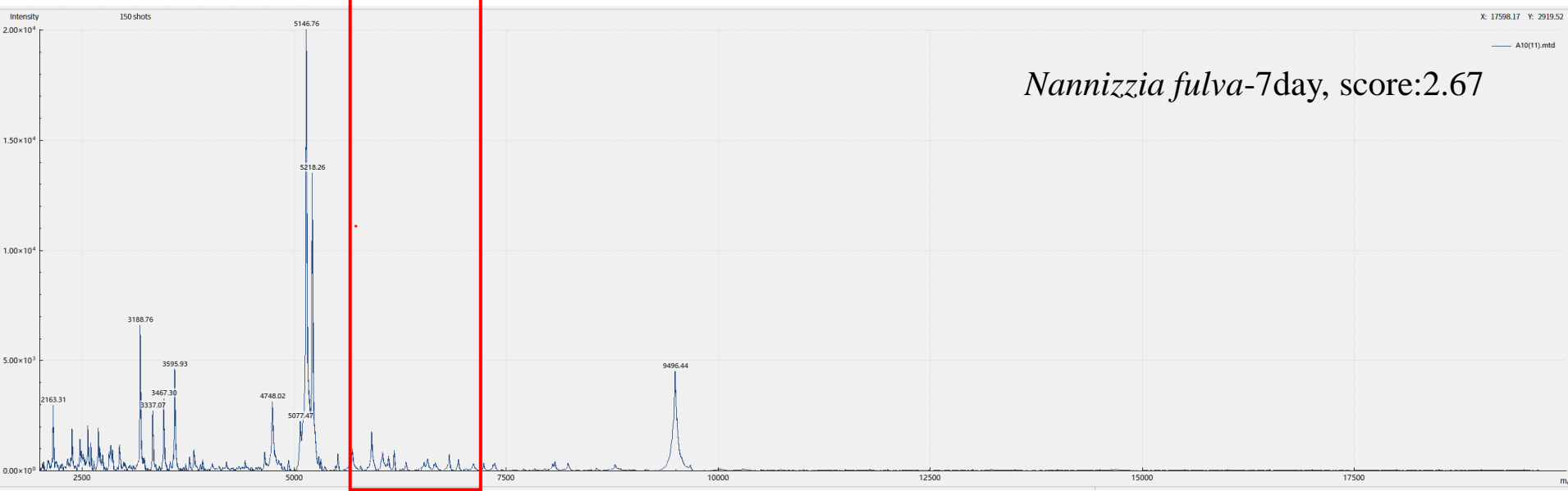

C

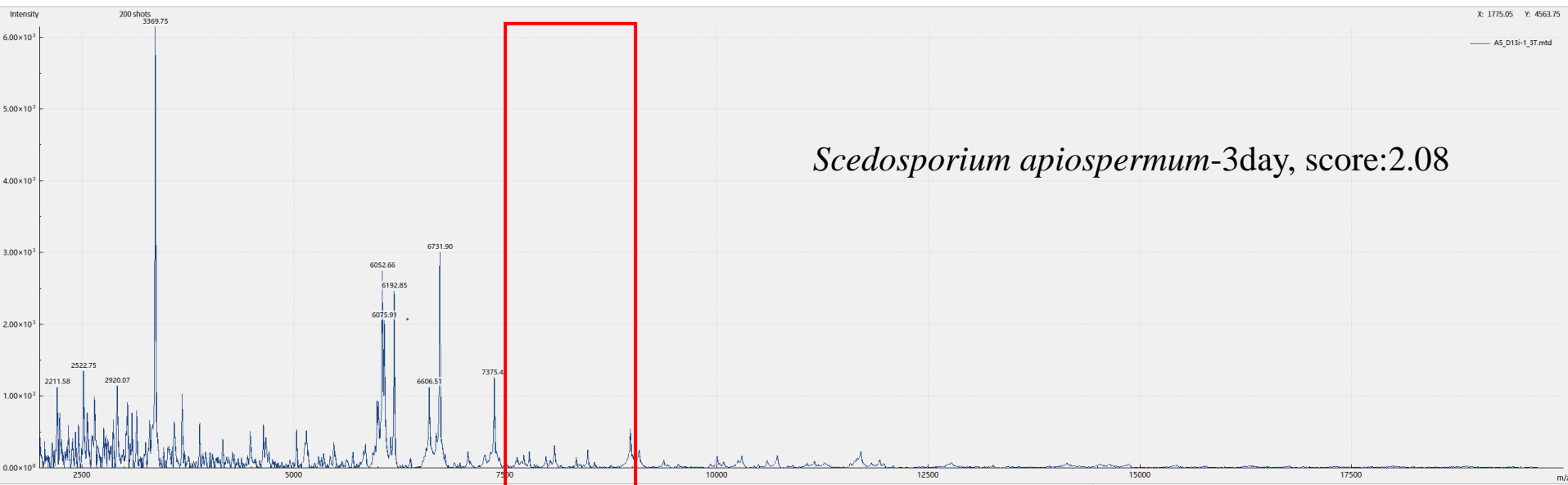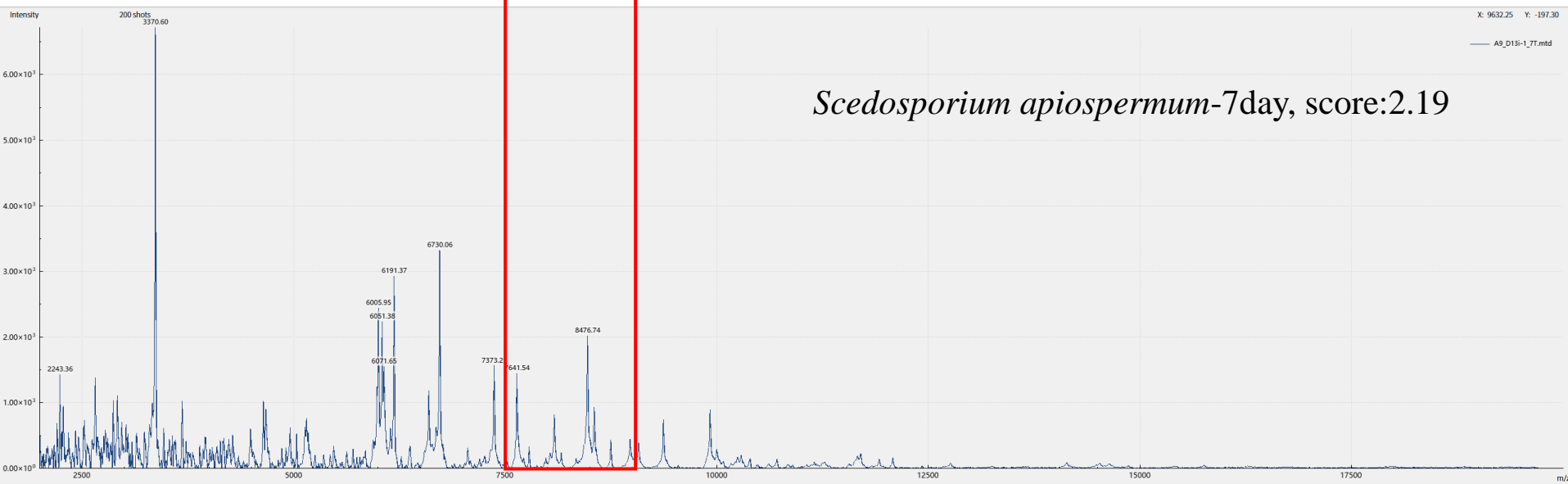

D

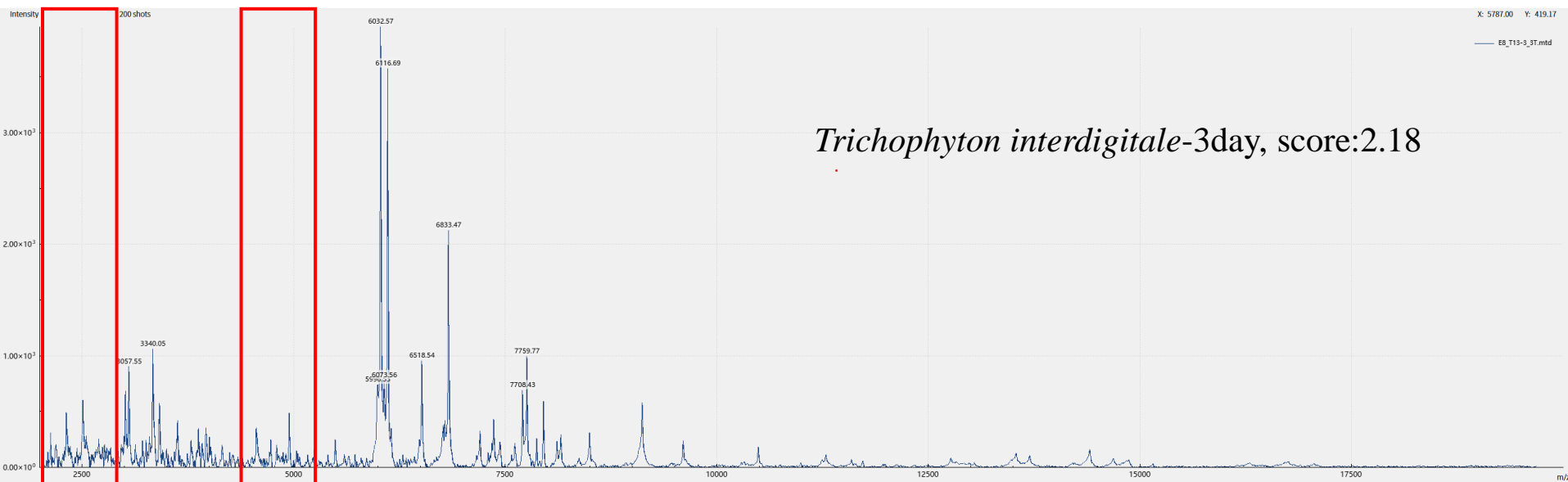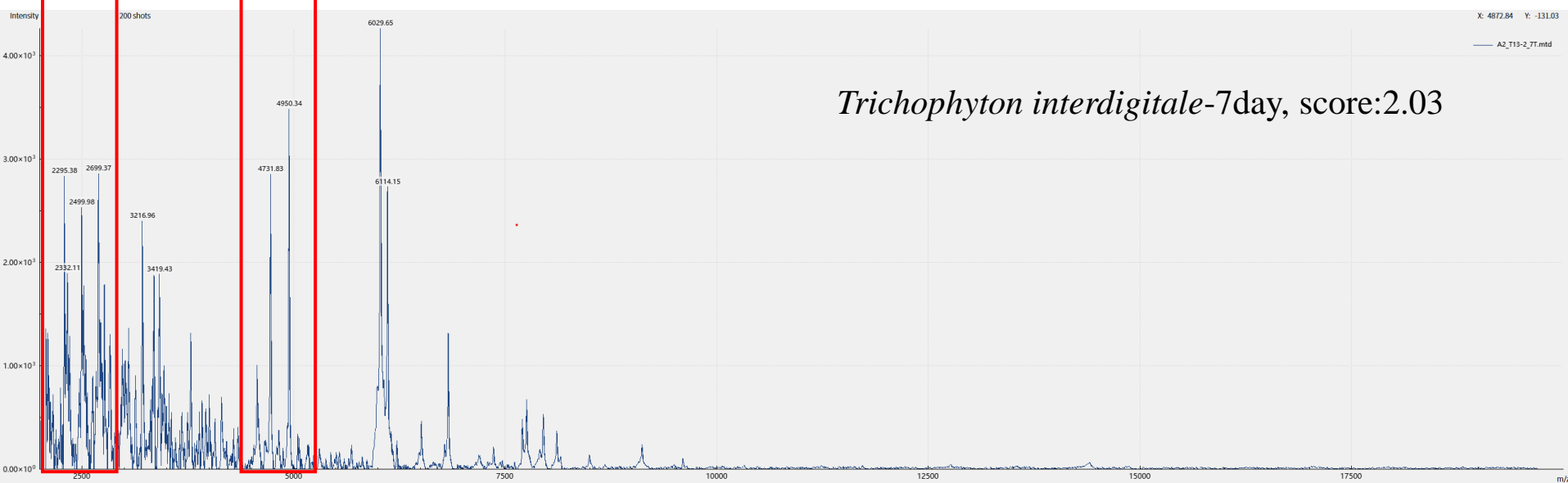

E

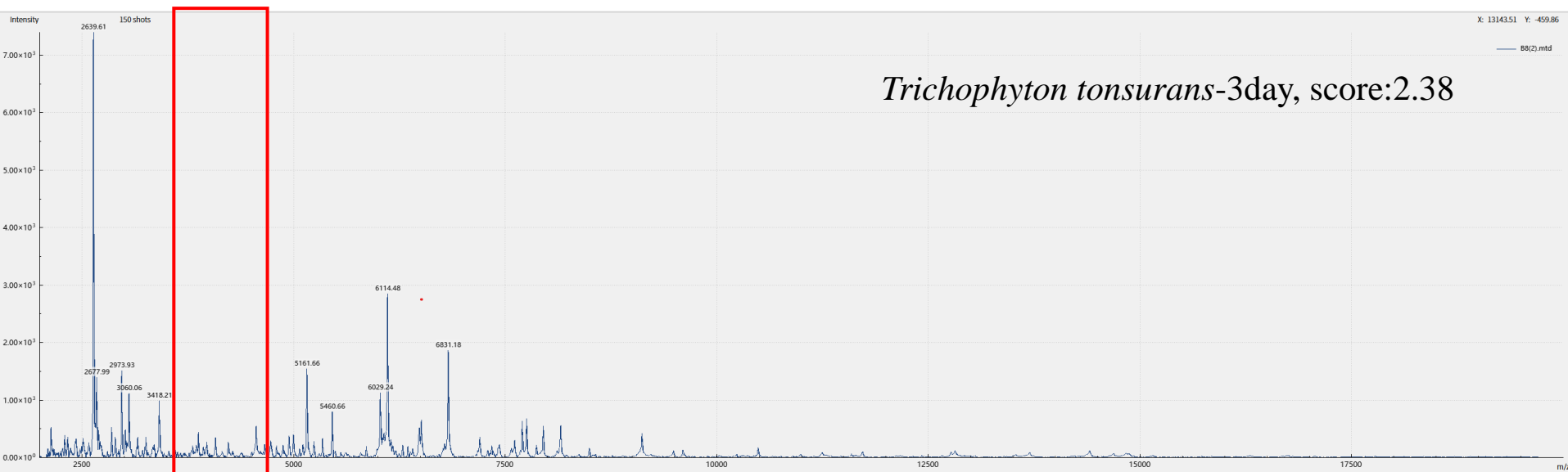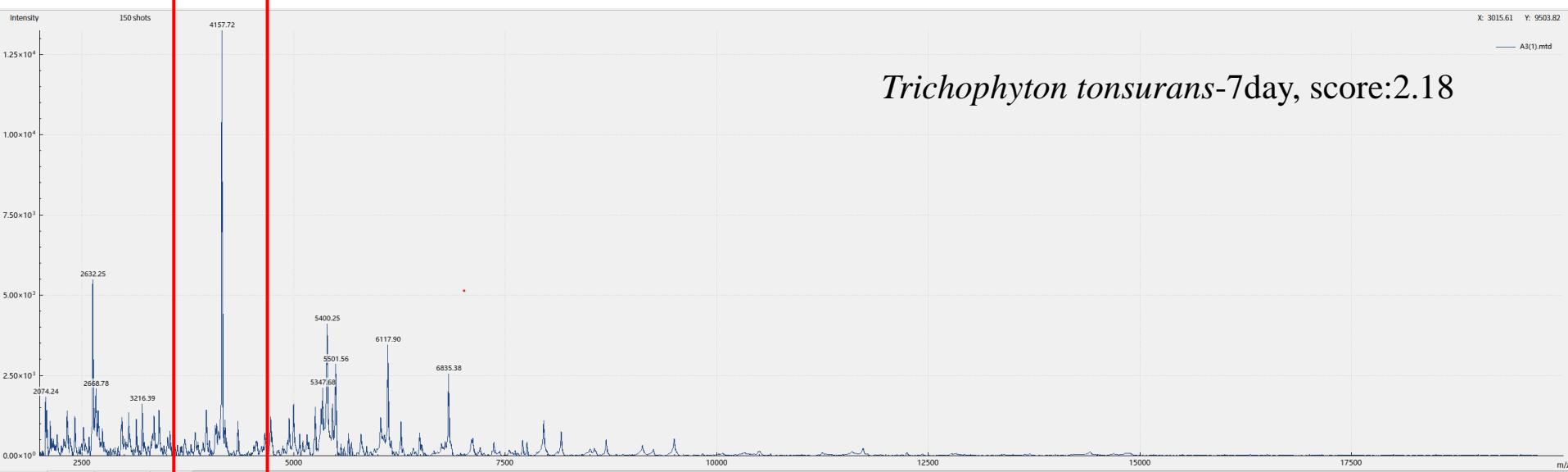

Supplement: Supplementary file 2 — Additional file 2: Supplementary Figure S1. Mass spectra of five representative filamentous fungi were collected at three and seven days of culture, and peaks with significant differences were marked in red boxes. [file 12866_2024_3394_MOESM2_ESM.pdf]
